# Smart Nanoparticles for Breast Cancer Treatment Based on the Tumor Microenvironment

**DOI:** 10.3389/fonc.2022.907684

**Published:** 2022-05-26

**Authors:** Xiao Luo, Qi Zhang, Hongbo Chen, Kai Hou, Ning Zeng, Yiping Wu

**Affiliations:** Department of Plastic and Cosmetic Surgery, Tongji Hospital, Tongji Medical College, Huazhong University of Science and Technology, Wuhan, China

**Keywords:** nanomaterial, nanotechnology, nanobiosensors, drug delivery, tumor microenvironment, smart nanoparticles

## Abstract

Breast cancer (BC) is the most common malignant tumor in women. There are different risk characteristics and treatment strategies for different subtypes of BC. The tumor microenvironment (TME) is of great significance for understanding the occurrence, development, and metastasis of tumors. The TME plays an important role in all stages of BC metastasis, immune monitoring, immune response avoidance, and drug resistance, and also plays an important role in the diagnosis, prevention, and prognosis of BC. Smart nanosystems have broad development prospect in the regulation of the BC drug delivery based on the response of the TME. In particular, TME-responsive nanoparticles cleverly utilize the abnormal features of BC tissues and cells to achieve targeted transport, stable release, and improved efficacy. We here present a review of the mechanisms underlying the response of the TME to BC to provide potential nanostrategies for future BC treatment.

## Introduction

Breast cancer (BC) is a common cancer that affects women’s health worldwide, and is also the most common malignant tumor among women (1, 2). According to the Report of the American Cancer Society, BC is second only to lung cancer in the number of cancer-related deaths among women. In recent years, the incidence of BC has been increasing at an annual rate of approximately 0.3%, with 2.3 million new cases worldwide in 2020 and a significant increase in mortality from 1990 to 2015 (3, 4). According to Surveillance, Epidemiology and End Results (SEER) data, the 5-year survival rate of patients with stage IIIA and stage IIIB BC was 52% and 48%, respectively, and the median survival of patients with stage III BC was 4.9 years (2, 5–8).

The histological types of BC are (1) non-invasive carcinoma, including ductal carcinoma *in situ* and lobular carcinoma *in situ*; and (2) invasive cancer, including no special type of invasive cancer, invasive lobular carcinoma, tubular carcinoma, and mucinous carcinoma (9–12). Based on molecular evidence, BC can be categorized into three groups: hormone receptor [estrogen receptor (ER) or progesterone receptor (PR)]-positive, human epidermal receptor 2 (HER2) or ERBB2 receptor-positive, and triple-negative BC (TNBC; ER−, PR−, HER2−). Among them, the luminal subtype can be further divided into luminal A and luminal B tumors, and TNBC can be further divided into six subtypes: basal-like1/basal-like2, mesenchymal, mesenchymal stem-like, immunomodulatory, luminal androgen receptor, and an unspecified group (11, 13–17). The luminal A subtype has the best clinical prognosis, accounting for 40% of all subtypes, and is characterized by high levels of ER expression; therefore, patients with luminal A BC are more likely to benefit from hormone therapy alone. Another less common subtype, luminal B BC (accounting for approximately 20% of all subtypes), is characterized by low levels of ER expression but high expression levels of proliferation-related genes and may require chemotherapy. HER2-enriched tumors (accounting for 15–20% of subtypes) are also characterized by overexpression of proliferation-related genes. The basal-like subtype is characterized by up-regulated expression of genes expressed in basal/myoepithelial cells. The specific molecular pathophysiological mechanism of TNBC tumors is still unclear, accounting for about 15% of all subtypes, and 71% of TNBC tumors are in the basal-like group (18–20).

The three main tumor subtypes of BC, characterized by ER or PR expression and amplification of the *ERBB2* gene, have different risk characteristics and treatment strategies, with the optimal treatment for each patient depending on the tumor subtype, anatomic tumor stage, and patient preference (11, 21–25). In addition, the role of genomic analyses such as detection of *BRCA1* or *BRCA2* germline mutations in treatment decisions has been shown to play an important role in clinical outcomes in BC (26–29).

For patients without metastatic disease, treatment aims to eradicate the tumor from the breast and regional lymph nodes and to prevent recurrence. According to the clinical setting, systemic therapy for non-metastatic BC is mainly based on the molecular characteristics (30–33): patients with hormone receptor-positive tumors receive endocrine therapy and a few patients also receive chemotherapy. Patients with ERBB2-positive tumors are typically treated with ERBB2-targeted antibodies or small-molecule inhibitors (trastuzumab-based) in combination with chemotherapy. Patients with TNBC tumors receive only chemotherapy (6, 14, 18). In addition, local treatment in patients with non-metastatic BC includes surgical excision (including axillary lymph node sampling or excision), and postoperative radiotherapy should be considered if lumpectomy is performed. However, the local treatment of patients with non-metastatic BC is increasingly being performed with some systemic therapy prior to surgery, such as neoadjuvant chemotherapy, followed by postoperative treatment tailored to the preoperative response (18, 34). In contrast, treatment for metastatic BC is also based on molecular subtypes, with the goal of prolonging life and reducing symptoms, which is more complex and personalized (7, 17). Metastatic BC also has a poor prognosis, with a median overall survival of approximately 1 year for metastatic TNBC, compared with approximately 5 years for the other two subtypes (8).

## The TME of BC

The proliferation of cancer cells and their spread to other parts of the body depend on the microenvironment of the organ, which is also known as the “seed and soil” hypothesis. In brief, cancer-associated fibroblasts (CAFs) in the tumor microenvironment (TME) promote metastasis and secrete large amounts of pro-inflammatory cytokines and chemokines, growth factors, and extracellular matrix (ECM) components. In addition to binding to adjacent tumor cells, CAFs interact with other stromal components in the microenvironment through complex crosstalk. These cells are involved in various activities such as angiogenesis and ECM remodeling, which contribute to tumor growth and metastasis. Thus, the TME is, to some extent, the protagonist of cancer progression, and is more genetically stable than the cancer itself. Therefore, increasing studies aiming toward anticancer drug development are currently focused on strategies targeting the TME (35–37). For example, owing to the genetic and phenotypic heterogeneity of BC cells, the cells or pathways vary among patients or at different periods in the disease course in the same patient, which poses a challenge in conducting targeted research for treatment (38). TME-targeted therapies that use tumor cells, key TME components, or secreted bioactive molecules alone or in combination as therapeutic targets are gaining increasing attention.

Here, we focus on research progress in the development of innovative nanomaterials delivery systems as the key to providing intelligent delivery systems based on active targeting and internalization in the TME and cancer cells (39, 40). In particular, the size and versatility of nano-loaded particles allow for chemical or genetic modification of their surfaces with various targeting groups or active ligands that trigger the specific orientation and recognition of biological targets, enabling them to deliver a steady amount of drugs at the tumor site and control their ability to deliver the drugs effectively (41–43). This review therefore provides an overview of the mechanisms of the TME response to BC, along with examples of the diversity of nanoparticle (NP)-based drug delivery systems developed to date or with potential to target the TME, which can offer an effective dual targeting strategy for BC cells and the TME as the potential key to realizing the successful treatment of BC (44, 45).

The TME is the internal environment in which tumor cells generate and live, which includes not only the tumor cells themselves but also fibroblasts, immune and inflammatory cells, glial cells, and other cells closely related to and interacting with tumor cells. The TME also includes interstitial cells, microvessels, and biomolecules such as secretory factors, proteins, and RNA in the surrounding area (46–49). The TME has long been a key focus of research and the core direction toward gaining a comprehensive understanding of the occurrence and development of tumors. In particular, the TME plays an important role in the transfer process, immune escape, and drug resistance in tumor metastasis, requiring immune monitoring of the TME at various stages (50) for the diagnosis, prevention, and prognosis of tumors (51–54).

Moreover, the TME plays a key role in several malignant phenotypes associated with tumor progression, including the acquisition of aggressive phenotypes, cell migration ability, chemotherapy resistance, protection from anti-tumor immune responses, and neovasculogenesis (46, 47, 55). The TME can also recombine with the ECM to enhance the permeability of chemotherapeutic drugs, as well as with NPs that enable the intelligent coupling of chemotherapy and immunotherapy by increasing immunogenicity and stimulating anti-tumor immunity (35).

## Smart NP-Based Systems for BC Treatment Based on the TME

Nanotechnology has developed rapidly in recent years, providing a good means to overcome the bottleneck problem of non-specific and non-selective damage to body tissues caused by traditional therapies (e.g., chemotherapy, radiotherapy and immunotherapy), and exhibiting unique advantages in improving the effects of drugs and radiation therapy while reducing adverse reactions (44, 56–58). On the one hand, multifunctional nanocarriers can selectively transport therapeutic drugs by utilizing the difference between the tumor tissue and normal tissue to enhance drug permeability and retention. On the other hand, a series of unique physical and chemical properties produced by the TME, such as weak acidity, a reducing environment, abnormal temperature gradient, overexpressed proteins and enzymes, and hypoxia, can be used to regulate the release of loaded drugs by nanocarriers (44, 59).

### Reactive Oxygen Species (ROS)-Responsive NPs

Dr. Otto Heinrich Warburg’s work on sea urchin embryology led to pioneering research on the physiological effects of ROS. His discoveries in the field of oxidative respiration earned him a Nobel Prize in 1931, and in 1926, Warburg also pioneered a model of tumor maintenance of oxidation and energy, hoping to exert a profound influence on cell physiology and metabolism by deeply exploring the basic properties of ROS (33).

ROS represent the products of single electron reduction of oxygen in the body, as a form of electron leakage before passing to the terminal oxidase respiratory chain, consuming approximately 2% of the oxygen generated, including the single-electron reduction product of oxygen superoxide anion (O2·^–^), two-electron reduction product hydrogen peroxide (H_2_O_2_), three-electron product hydroxyl free radical (·OH), and nitric oxide, among other highly reactive agents (60). Intracellular ROS are by-products of mitochondrial processes, metabolism, and enzyme activity. Under normal physiological conditions, a large number of electrons are released and reduced by oxygen molecules in the high-oxygen environment and the highly reduced respiratory chain of mitochondria during the transition from state III to state IV (31).

Under normal physiological conditions, ROS can be effectively neutralized by the enzymes superoxide dismutase, catalase, glutathione, and thioredoxin to control the equilibrium oxidation-reduction (redox) state. However, when redox homeostasis is destroyed or relieved, changes in ROS levels will lead to physiological and pathological changes (61, 62). In addition, ROS play a positive role in regulating intracellular signal transduction pathways, with numerous biological conduits being constrained by a variety of ROS species, which are the key to maintaining cellular homeostasis. Any skew in this delicate balance is accompanied by a wave of events that can have either negative or positive impacts for the cell.

From the perspective of cancer, the conventional view that ROS cause widespread damage and chaos within cells has long been debated in the scientific community (63–65). Recently, great progress has been made in the understanding of ROS biology, and there is increasing evidence that the effects of ROS are gradient-dependent, with a threshold of habituation, which affects their signal transduction and toxicity induction function, thus resulting in either “good” or “bad” ROS (33). For example, ROS play a key role in the activation of tumor-promoting signaling pathways (64, 66, 67). Moreover, abnormal production of ROS and ineffective neutralization of excessive ROS levels can lead to tumor growth and progression through different signaling pathways, including phosphatidylinositol 3-kinase/protein kinase inhibitor/mammalian target of rapamycin (PI3/Akt/mTOR), vascular endothelial growth factor (VEGF)/VEGF receptor, phosphatase and tensin congeners (PTEN), and matrix metalloproteinase (MMP) pathways (68). Therefore, an intelligent collaborative treatment system can be developed to ensure the release of chemotherapeutic drugs on demand and reduce ROS production.

### pH-Responsive NPs

The acidic microenvironment caused by massive anaerobic glycolysis is one of the important characteristics of malignant tumors, and is also an important factor inducing the occurrence, metastasis, and drug resistance of BC. In recent years, with increasing understanding of the acidic TME, it has been considered a new target for tumor diagnosis and treatment, which is of great significance for the design of pH-responsive nanomedicine and nano-diagnosis (69).

Gong et al. (70) developed a pH-responsive co-delivery platform for metformin and small interfering RNA (siRNA) targeting the immune checkpoint inhibitor FGL1 (siFGL1) based on hybrid biomimetic membrane-camouflaged poly (lactic-co-glycolic acid) NPs. This pH-triggered CO_2_ gas generation nanoplatform was developed using the metformin guanidine group, which can react reversibly with CO_2_ to produce a metformin-CO_2_ complex. Moreover, the capture/release of CO_2_ depends on the pH value, thus improving the *in vivo* lysotic escape of the siRNA to achieve the cytoplasmic transfer of siRNA. Metformin promotes programmed death ligand 1 (PD-L1) by activating adenosine monophosphate-induced protein kinase degradation, and then blocks the inhibitory signal of PD-L1, while siFGL1 can silence the *FGL1* gene, promote the T cell-mediated immune response, and enhance anti-tumor immunity. The combination of these two drugs exhibited a high synergistic therapeutic effect on BC *in vivo* and *in vitro* (70).

In addition, Shen et al. (71) developed a robust nanoplatform fabricated from a TME pH-responsive polymer (MEO-PEG-B-PPMEMa) and a cationic lipid compound (G0-C14) for the *in vivo* delivery of cytotoxic saponins in BC treatment. The nanoplatform responds to the TME-specific pH with the rapid release of the saporin/G0-C14 complex, which significantly enhanced the cytoplasmic uptake of saporin and subsequent intracellular escape by tumor cells, thereby effectively inhibiting tumor growth (71).

### Enzyme-Responsive NPs

Enzymes play an important role in metabolism. Thus, abnormal metabolism in the TME will result in the overexpression of certain enzymes. Typical examples are MMPs, which are calcium- and zinc-dependent endopeptidases secreted by cancer cells, tumor blood vessels, and CAFs, and play a key role in cancer cell ECM remodeling (72). Other overexpressed enzymes in the TME include hyaluronidase and lysyl oxidase, along with other enzymes that are overexpressed in specific tumors (73).

Kashyap et al. (74) synthesized a hydrophobic acrylate monomer from the natural resource 3-pentachylphenol by enzyme and thermal reactions, which was co-polymerized with the hydrophilic monomer polyethylene glycol acrylate. This copolymer core-shell NP system was then used for the extracellular and intracellular delivery of doxorubicin (DOX)-responsive polymer nanoscaffolds to improve cancer treatment. In the presence of esterase (pH = 7.4, 37°C), the amphiphilic copolymer broke down in a slow and controlled manner within 12 h, releasing >95% of the drugs and achieving controlled release of the drugs in cells (74). For breast cancer, MNPs were successfully conjugated with MTX by Nosrati et al. and this drug delivery system is dependent on the release of the MTX within the lysosomal compartment for BC cells treatment (75).

### Hypoxia-Activated NPs

The partial pressure of oxygen (PO_2_) in various tumors is generally below 60 mmHg and gradually decreases with tumor progression. This reduction mainly occurs because the structure and function of tumor microvessels are abnormal, and the diffusion distance between blood vessels and the tumor cells increases, thereby preventing the effective transport of O_2_ to specific sites. Moreover, the reduced blood oxygenation capacity due to tumor growth or therapeutic anemia also contributes to the local PO_2_ reduction (72). The resulting hypoxic environment not only promotes tumor growth, progression, metastasis and damage normal tissues but also reduces the efficacy of radiotherapy and chemotherapy (76).

Mpekris et al. (77) found that the blood vessels of lung metastases of BC were compressed, leading to hypoxia. Based on this principle, mechanical tranilast therapy was used to decompress the blood vessels of lung metastasis in mice so as to restore perfusion and relieve hypoxia. This strategy enabled the immune checkpoint blocker atezolizumab and the nanodrug doxil to more effectively exert cytotoxic effects on metastases and stimulate an antitumor immune response (77).

### Thermo-Responsive NPs

Compared with normal tissue, the tumor tissue has a higher temperature, which makes it possible to control and release drugs by external heating at the tumor site, and this property has been exploited in the development of smart drug delivery systems (SDDSs) targeting tumors (74, 78–82). Ding et al. (79) designed a multifunctional SDDS with a double-layer structure: a thermo-sensitive copolymer as the building block of the core-shell structure, two chemotherapeutic drugs [paclitaxel (PTX) and DOX; NP-PD) were encapsulated to form copolymers, and the siRNA (NP-PD-S) affecting survival was absorbed on the surface to form nanostructures, which were finally wrapped by self-polymerizing dopamine (PDA) membranes (NP-PD-S-PDA). This PDA film not only exhibited a photothermal therapeutic effect but also had a protective effect against the uncontrolled release of the drugs. *In vitro* experiments with the MDA-MB-231 BC cell line showed that the survival rate of NP-PD-S-PDA was significantly decreased after 5 min of laser irradiation, indicating that this is an effective combination therapy. *In vivo* experiments with female BALB/C nude mice also showed that in tumors irradiated by NP-PD-S-PDA plus near infrared light, PDA generated sufficient heat, and thermal ablation led to collapse of the NPs, thereby triggering the release of PTX and DOX within the tumor to ultimately achieve antitumor effects (79).

In addition, Hu et al. (80) developed a system for delivery of the hydrophobic chemotherapy drug PTX and the biological macromolecule interleukin (IL)-12 through the low-temperature expansion effect of the acid-sensitive material MPEG-Dlinkm-PDLla and pluronic F127 co-delivered NPs. PTX and IL-12 jointly activated T lymphocytes and natural killer cells to release interferon-gamma, selectively inhibited regulatory T cells, induced the M1-type differentiation of tumor-associated macrophages, improved the tumor immunosuppressive microenvironment, significantly inhibited the growth and metastasis of 4T1 BC cells, and prolonged the overall survival of tumor-bearing mice (80).

### CAF-Regulating NPs

The tumor microenvironment is the ecosystem that surrounds a tumor inside the body. It includes immune cells, the extracellular matrix, blood vessels and other cells, like fibroblasts. CAFs are the most abundant cells in the TME matrix, secreting a large number of growth factors (e.g., transforming growth factor-β, VEGF, platelet-derived growth factor), cytokines, interleukins, chemokines, and ECM proteins (e.g., MMPs), which all have an impact on tumor development. CAFs actively reconstruct the cellular and stromal components of the TME. M2-polarized macrophages promote metastasis and contribute to the development of an immunosuppressive environment. Endothelial cells and pericytes contribute to tumor angiogenesis and handle the oxygen supply. The interstitial ECM supports the tumor structure and regulates drug penetration. The external matrix basement membrane acts as a barrier around the tumor. Both the external matrix and blood vessels contribute to increased interstitial fluid pressure and tumor hypoxia, which is a major barrier to tumor treatment. MMP also supports tumor growth, creates physical barriers to drugs and immune invasion, and promotes cancer invasion (83, 84). In short, normal fibroblasts inhibit tumor formation, whereas CAFs promote BC cell proliferation, angiogenesis, and the inflammatory response, and remodeling the ECM promotes the malignant phenotype of BC cells, resulting in a poor prognosis (85, 86).

Some biological markers of CAFs can be used to identify and target CAFs in the BC TME, which creates opportunities for nanodelivery systems to specifically target the BC metastatic TME. These proteins include fibroblast activating protein (FAP), alpha-smooth muscle actin, fibroblast-specific protein, vimentin, and proline 4-hydroxylase (87, 88). As the most common tumor-targeting CAF antigen, FAP is overexpressed on the surface of CAFs, and nanoparticles can specifically target FAP to promote the infiltration and accumulation of nano-loaded drugs in tumor tissues, thus playing an anti-tumor role. Zhen et al. (89) developed a photoimmunotherapy nanostrategy using ferritin as a photosensitizer carrier and a single-chain variable fragment of anti-FAP antibody conjugated with ferritin.

## Conclusions and Perspectives

There has been great progress in the development of intelligent NPs for the effective loading of anti-BC drugs, specific enrichment of tumor cells and tissues, and the controlled release and efficient delivery of drugs. In particular, regulation based on the response of the TME including multifunctional nanoparticles has a broad development prospect for improving BC drug delivery. TME-responsive NPs cleverly utilize the abnormal features of BC tissues to achieve targeted transport, stable release, and improved drug efficacy.

However, there is still room for improvement in TME-responsive nanotechnology. First, there are limitations of the nanomaterials themselves, including limited understanding of the mechanism underlying the interaction between materials and other systems in the body, which makes clinical translation difficult. In addition, the interaction and influence of different components in the nanosystem represent important questions that have not yet been elucidated. Therefore, the design of improved NPs including multifunctional NPs is a promising direction in the future.

## Author Contributions

All authors contributed to the design of the study and writing of the manuscript. XL, KH, and HC undertook the research, YW and ZN wrote the main manuscript text and prepared figures. QZ and YW revised the article critically for important intellectual content and final approval of the version to be submitted. All authors reviewed the manuscript.

## Conflict of Interest

The authors declare that the research was conducted in the absence of any commercial or financial relationships that could be construed as a potential conflict of interest.

## Publisher’s Note

All claims expressed in this article are solely those of the authors and do not necessarily represent those of their affiliated organizations, or those of the publisher, the editors and the reviewers. Any product that may be evaluated in this article, or claim that may be made by its manufacturer, is not guaranteed or endorsed by the publisher.

## Abbreviations

BC, breast cancer; TME, tumor microenvironment; HER2, human epidermal growth factor receptor 2; ER, estrogen receptors; PR, progesterone receptors; TNBC, triple negative breast cancer; CAFs, cancer-associated fibroblasts; ECM, extracellular matrix; NPs, nanoparticles; ROS, reactive oxygen species; DOX, doxorubicin.
